# Kinesiophobia and related factors in systemic lupus erythematosus patients

**DOI:** 10.3906/sag-1804-152

**Published:** 2019-10-24

**Authors:** Songül BAĞLAN YENTÜR, Saliha KARATAY, Deran OSKAY, Abdurrahman TUFAN, Hamit KÜÇÜK, Şeminur HAZNEDAROĞLU

**Affiliations:** 1 Department of Physiotherapy and Rehabilitation, Faculty of Health Sciences, Gazi University, Ankara Turkey; 2 Department of Physical Medicine and Rehabilitation, A Life Park Hospital, Ankara Turkey; 3 Division of Rheumatology, Faculty of Medical Sciences, Gazi University Turkey; 4 Erzurum Regional Education Hospital, Erzurum Turkey

**Keywords:** Systemic lupus erythematosus, kinesiophobia, physical activity

## Abstract

**Background/aim:**

This study was designed to investigate the relationship between kinesiophobia and the level of physical activity, depression, disease activity, fatigue, pain, and quality of life in female patients with systemic lupus erythematosus (SLE).

**Materials and methods:**

Seventy volunteer female patients were included in the study. Kinesiophobia, physical activity level, disease activity, fatigue, depression, pain, and quality of life were assessed using the Tampa Scale for Kinesiophobia (TSK), International Physical Activity Questionnaire- Short Form (IPAQ), Systemic Lupus Erythematosus Disease Activity Index (SLEDAI), Fatigue Severity Scale (FSS), Beck Depression Inventory (BDI), McGill Pain Questionnaire- Short Form (MPQ-SF) and Nottingham Health Profile (NHP), respectively.

**Results:**

Two-thirds of the patients in the study had a high degree of kinesiophobia. Although there was a significant correlation between kinesiophobia and depression and some subscales of quality of life (sleep, social isolation, emotional reactions) (P < 0.05), no significant correlation with other parameters was found.

**Conclusion:**

As a result of this study, the majority of SLE patients included in the study were identified as having high levels of kinesiophobia. Patients’ fear and avoidance reaction from movement can be influenced by psychosocial factors. Treatments focusing on kinesiophobia of SLE patients could be beneficial in increasing the success of rehabilitation.

## 1. Introduction

Systemic lupus erythematosus (SLE) is a chronic, inflammatory, autoimmune, and rheumatic disease causing damage to almost all internal organs and also causing various clinical symptoms by autoantibodies that bind to the tissue and immune complexes. The etiology of SLE is still unknown. However, a variety of factors such as genetic factors, environmental factors, and immunological disorders are considered to be responsible [1]. Women are affected nine times more than men. The disease shows a significant increase in women within the productive term. The clinical course of the disease is characterized by consecutive periods of activation and remission. Disease-specific autoantibodies are revealed in the autoimmune phase. Disease-related damage can occur in this phase [2].

The clinical presentations of the disease shows a wide spectrum. As mild symptoms can be observed, severe, and even life-threatening symptoms can also be encountered [1]. The most common symptoms are observed in the skin, musculoskeletal system, renal system, nervous system, cardiovascular system, and pulmonary system, and can also be hematologic [3]. One of the most common presentations is encountered in the musculoskeletal system. Many different manifestations like; arthralgia, arthritis, myalgia, tenosynovitis, tendon ruptures, and muscle weakness can be seen in the musculoskeletal system. Arthritis and arthralgia are observed in more than 95% of patients and are among the initial symptoms in approximately 50% of patients. Tenosynovitis is one of the early symptoms in SLE patients. Myalgia and muscle weakness can also be seen in the overall inflammation period [4]. Arthralgia and arthritis observed in SLE patients may lead to the avoidance of physical activity over time [5]. Reduced physical activity levels have been identified in other chronic, inflammatory diseases such as rheumatoid arthritis (RA), primary Sjögren’s syndrome (SS), and ankylosing spondylitis (AS), as well as in patients with SLE [6]. In previous studies, it was reported that the level of physical activity can also be affected by fatigue, psychological status, disturbed sleep quality, and fear of movement in addition to pain and disease activity [6]. These factors also have a significant negative effect on SLE patients. Peripheral nervous system and central nervous system symptoms are commonly encountered in SLE and psychiatric disturbances can also be seen. Higher rates of depression and anxiety are observed in patients with SLE [7]. Fatigue, which is a frequently observed complaint of patients with SLE and depression, may together be the cause of activity avoidance in patients. Moreover, the fear of movement referred to as kinesiophobia can lead to physical inactivity [5].

Kinesiophobia is defined as excessive and irrational fear against physical activity and exercise after painful conditions or injuries. The role of fear of movement in physical inactivity has been demonstrated in acute and chronic musculoskeletal problems like low back pain and several rheumatic diseases such as primary SS and fibromyalgia [8,9]. Kinesiophobia can lead to physiological problems such as reductions in mobility and strength, and decrease in aerobic capacity. Avoidance of activities can lead to restrictions in the performance of activities and hinder correct activity. In chronic rheumatic disorders, arthritis, joint pain, and severe systemic involvement together give rise to kinesiophobia [5]. In previous studies, a high level of kinesiophobia was found amongst patients with RA and primary SS [9,10]. Despite the detection of low physical activity levels in SLE in various studies, there are very few studies which investigate the existence of kinesiophobia [11]. According to the authors’ knowledge, there are no studies of patients with SLE which investigate the level of physical activity and factors affecting the degree of kinesiophobia. Therefore, the aim of this study is to investigate the relationship between kinesiophobia with physical activity level, depression, disease activity, fatigue, pain, and quality of life in patients with SLE. We hypothesized that there is a significant correlation between kinesiophobia and other parameters in patients with SLE.

## 2. Materials and methods

### 2.1. Patients

This study was carried out in accordance with the Helsinki Declaration and was approved by the local ethics committee of Gazi University. A written and signed consent form was taken from each volunteer. 

All patients under follow-up were included. No sample size calculation was done. Eighty patients with SLE were invited to join the study (Figure). The invited patients were registered for follow-up at Gazi University Division of Rheumatology outpatient clinic with a diagnosis of SLE. Patients who were pregnant, were diagnosed with malignancy, had changes of medical treatment in the last three months, had dysfunction that limited physical activity such as severe neurological impairment, immobility or cooperation deficits were excluded from the study. Age, body mass index (BMI), occupation, smoking/alcohol habits, any kind of corticosteroid drug use and duration of the disease were recorded. Whether patients had an exercise habit or not was also recorded.

**Figure 1:**
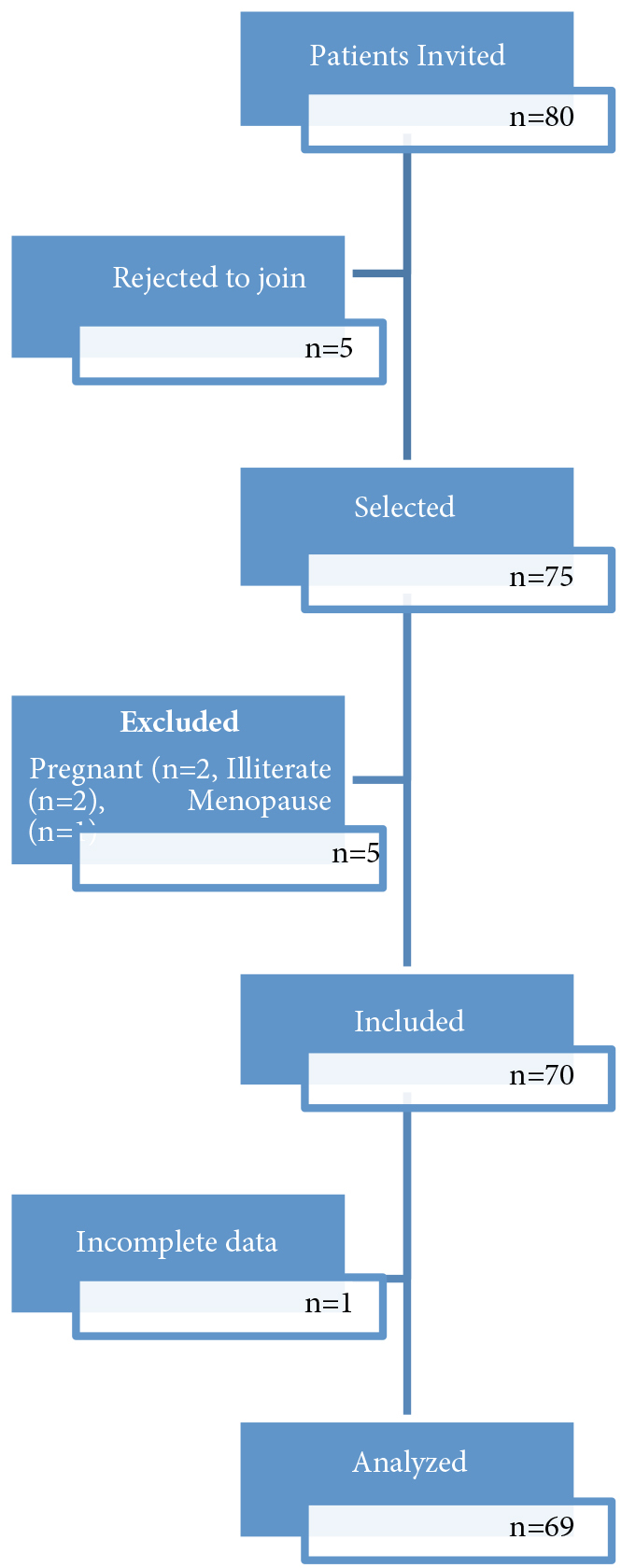
Patient flow diagram.

### 2.2. Methods

#### 2.2.1. Kinesiophobia

To determine the level of kinesiophobia, Tampa Kinesiophobia Scale-Turkish version was used. This scale consists of 17 questions which assess pain or fear and avoidance associated with past injury. Four-point Likert scales were used for the evaluation of the questions (1 = I strongly disagree, 2 = disagree, 3 = agree, 4 = strongly agree) [12]. Vlaeyen et al. describe 37 points or more as high kinesiophobia score on this scale [13]. 

#### 2.2.2. Physical activity level

The Turkish version of the International Physical Activity Questionnaire - Short Form was used to assess the physical activity level (IPAQ-SF). This questionnaire obtains information about how much time is spent while walking and in moderate and vigorous activities in the last seven days. There is also a separate section about sitting duration [14]. 

#### 2.2.3. Fatigue

Fatigue was assessed via the Turkish version of Fatigue Severity Scale (FSS). This scale consists of nine questions; each question is scored from one to seven [15]. 

#### 2.2.4. Depression

In order to assess the level of depression in patients, the Turkish version of Beck Depression Inventory (BDI) was used. BDI consists of 21 items related to depressive symptoms such as pessimism, sense of failure, guilt, dissatisfaction, sleep loss, appetite loss, and fatigue. Each item is scored between 0 and 3. Total score is 63, and an increase of the score means increased severity of depression. According to this scale 1–10 points are defined as normal, scores between 11 and 16 indicate mild mental distress, 17–20 indicate that the patient is at the border points for clinical depression, 21–30 indicate moderate depression, severe depression scores are between 31 and 40, and 40 and above are interpreted as very serious depression [16]. 

#### 2.2.5. Pain

The type and severity of pain were evaluated with the Short Form McGill Pain Questionnaire. This questionnaire consists of 11 sensory and 4 affective descriptive words. These 15 words are described as 0 = absent, 1 = mild, 2 = moderate, 3 = severe pain. Thus, three types of pain scores (sensory, affective, total = sensory + affective) are obtained. In McGill Pain Questionnaire, the current level of pain is measured by Visual Analog Scale (VAS) and Likert scale consisting of 6 points (0 = no pain, 1 = mild, 2 = irritating, 3 = bothersome, 4 = terrible, 5 = unbearable) [17]. 

#### 2.2.6. Disease activity

Disease activity was assessed with the Turkish version of Systemic Lupus Erythematosus Disease Activity Index (SLEDAI). SLEDAI was administrated by a physician at Gazi University Rheumatology clinic. The most common and important 24 symptoms associated with the disease are included in this index. Items affecting vital functions are scored higher points. Total score is 105, so patients can receive a score between 0 and 105 [18]. 

#### 2.2.7. Quality of life

The quality of life was evaluated by Nottingham Health Profile (NHP). NHP is a questionnaire, assessing emotional, physical, and social problems. There are six subscales consisting of 38 questions in total. These subscales are pain (8 questions), physical activity (8 questions), fatigue (3 questions), sleep (5 questions), social isolation (5 questions), and emotional reactions (9 questions). Patient is asked to answer the questions as a yes or no. The total score for each section is 100. Increase in score corresponds to a decrease in the quality of life [19].

### 2.3. Statistical analysis

Statistical analysis was performed using IBM SPSS version 20 software. Normal distribution of the data was checked using the Kolmogorov–Smirnov test. All variables were normally distributed. Statistically significant correlation coefficients were calculated using Pearson’s correlation test. Independent samples t-test and one-way ANOVA were used for bivariate comparisons. Linear regression analysis was performed to look for factors effecting kinesiophobia scores. Type-1 error level of 5% was used for statistical significance.

## 3. Results

Seventy female patients with SLE aged between 18 and 55 years were included in the study. Results of one patient were not analyzed because of failing to complete questions. Hence, the study was concluded with 69 patients. When the physical characteristics of the patients were evaluated, we found that the mean age was 37.50 ± 9.56 years, the mean BMI was 25.68 ± 5.16 kg/m², and the average duration of disease was 7.85 ± 6.68 years. Descriptive characteristics of the patients are shown in Table 1.

**Table 1:** Descriptive characteristics of the patients.

	n (%)
OccupationHousewife Employed	53 (76.8)16 (23.2)
Smoking habitsYes No	18 (26.1)51 (73.9)
Alcohol habitsYes No	0 (0)69 (100)
Using corticosteroidsYes No	33 (47.8)36 (52.2)
Diabetes Mellitus Yes No	1 (1.4)68 (98.6)
HypertensionYes No	60 (87)9 (13)
Family history for rheumatic diseaseYes No	26 (37.7)43 (62.3)
Regular exercise habitsYes No	9 (13)60 (87)

Classifications of participants according to their kinesiophobia, physical activity, and depression levels are shown in Table 2.

**Table 2:** Kinesiophobia, physical activity, and depression levels of participants.

	n (%)
Kinesiophobia Low High	21 (30.4)48 (69.6)
Physical activity Inactive Moderate active Adequately active	36 (52.2)28 (40.6)5 (7.2)
Depression scores Normal Mild depression Borderline depression Moderate depression Severe depression Very severe depression	32 (46.4)22 (31.9)3 (4.3)7 (10.1)4 (5.8)1 (1.5)

Mean data relating to other variables of SLE patients participating in our study are shown in Table 3.

**Table 3:** Mean data for fatigue, disease activity, pain, and quality of life in patients with SLE.

Variables	X ± SD (n = 69)
FSS	38.07 ± 16.63
SLEDAI	9.48 ± 5.20
MPQ (Sensory words score)	3.43 ± 5.13
MPQ (Afective words score)	0.81 ± 1.49
MPQ (Total score of sensory and affective words)	4.21 ± 6.34
MPQ (VAS score)	3.30 ± 3.78
MPQ (Likert score)	1.60 ± 1.86
NHP (Pain subscale)	0.36 ± 0.38
NHP (Physical activity subscale)	0.33 ± 1.29
NHP (Fatigue subscale)	0.35 ±0.44
NHP (Sleep subscale)	0.29 ±0.31
NHP (Social isolation subscale)	0.16 ±0.29
NHP (Emotional reactions subscale)	0.21 ±0.26

FSS: Fatigue Severity Scale, SLEDAI: Systemic Lupus Erythematosus Disease Activity Index, MPQ: McGill Pain Questionnaire, NHP: Nottingham Health Profile, SD: Standard Deviation

Among the variables studied, only depression scores showed a significant relationship with kinesiophobia scores in bivariate comparisons (Table 4).

**Table 4:** Differences in mean kinesiophobia scores with regard to different variables.

	Mean ± SD	t/F; P
OccupationHousewife (n = 53)Employed (n = 16)	40.2 ± 7.539.5 ± 6.8	t = 0.309; P = 0.758
Tobacco useSmoker (n = 18)Non-smoker (n = 51)	40.1 ± 8.039.9 ± 5.2	t = −0.074; P = 0.941
Drug useYes (n = 33)No (n = 36)	39.9 ± 7.340.1 ± 7.5	t = −0.065; P = 0.948
Body mass indexNormal (n = 35)Overweight (n = 18)Obese (n = 16)	40.5 ± 8.139.9 ± 6.339.0 ± 6.8	t = 0.231; P = 0.794
DepressionNormal (n = 32)Mild (n = 22)Moderate (n = 10)Severe (n = 5)	38.5 ± 7.538.9 ± 5.142.6 ± 6.849.2 ± 9.6	F = 4.155; P = 0.009
Physical activityLow (n = 36)Moderate (n = 28)Adequate (n = 5)	40.639.041.4	F = 0.487; P = 0.617
HypertensionPresent (n = 9)Absent (n = 60)	39.9 ± 4.440.0 ± 7.7	t = 0.048; P = 0.962

### 3.1. Factors affecting kinesiophobia

When the correlation between kinesiophobia scores and other factors was investigated, kinesiophobia had a significant relationship with depression (P = 0.005; r = 0.335) and certain subscales of quality of life (sleep (P = 0.0044; r = 0.245), social isolation, (P = 0.003; r = 0.350), and emotional reactions (P < 0.001; r = 0.435)). Other investigated variables had no correlation with the kinesiophobia scores (P > 0.05).

A linear regression model was built to check for variables independently effecting kinesiophobia scores. The following numerical variables were entered into the model: depression scores, BMI, duration of medication, FMS, fatigue, physical activity score, disease activity, pain score, and age. The stepwise analysis showed that depression scores were the only significant factor independently effecting kinesiophobia scores (R2 = 11.0%; Beta = 0.255 95% CI for Beta [0.072–0.437]; P = 0.007).

## 4. Discussion

This study is the first study evaluating kinesiophobia and related factors in patients with SLE. According to the results of the study, 66.6% of patients were identified as having high levels of kinesiophobia. There was a strong relationship between kinesiophobia and depression. Moreover, significant associations between kinesiophobia and sleep, social isolation, and emotional reactions subscales of quality of life were observed. In addition, 53.63% of these patients were determined to range from mild to very severe depression levels. Depression level had a significant relationship with all other parameters evaluated except physical activity levels and subparameters of physical activity of quality of life.

Kinesiophobia is associated usually with several painful conditions, prerevised injury or surgery [8]. Fear that leads to activity avoidance can give rise to physiological disorders, such as decreased mobility, strength and aerobic capacity. Increased activity restriction may cause deterioration of the decreases in activity tolerance and stability. Fear and anxiety can lead to increased pain perception [10]. In chronic rheumatic diseases like SLE, severe joint complaints such as arthritis and arthralgia may occur as well as life-threatening manifestations [4]. These symptoms can particularly cause kinesiophobia at chronic process. In rheumatic diseases, studies of the presence of kinesiophobia are increasing day by day. In studies, the degree of kinesiophobia in patients with RA and SS was investigated using a variety of surveys. These studies have generally reported a high level of fear of movement [9,10]. When literature is examined, there are few studies evaluating the reluctance against movement and fear of movement in the course of patients with SLE [11]. Mancuso and his colleagues investigated the causes of physical inactivity in patients with SLE by asking open-ended questions. As a result, general obstacles and disease-related blocks were reported as barriers involved in physical inactivity. At the same time, the authors have determined that patients do not want to be active because they feared an increase in symptoms. Of patients, 78% do not want to move because of the pain and fatigue, and also stated that these symptoms discourage them. For these reasons, even if they begin physical activity, they stated that they do not want to continue. However, 92% of the patients stated that they should be more active and they believe in the benefits of physical activity [11]. In this study, fear of movement in patients was determined with open-ended questions asking their views on the issue rather than a reliable survey. Therefore, it was not possible to score and rate the fear of movement the patients were undergoing. However, the researchers have set themselves some questions which help them having yes/no answers from patients on the form and created a percentage of the given answers. There is only one study evaluating kinesiophobia in SLE with scales. Cezarino et al. used TSK to assess kinesiophobia and found 42 points as mean value, which shows a high score. However, they did not classify the patients according to scores and did not determine the level of relationship between various factors that may affect kinesiophobia [20]. In our study, we used TSK, one of the two scales evaluating pain and fear associated with injury. Our assessment of TSK has enabled us to both transfer kinesiophobia to digital data with an objective form, and rate this information. 

The association between pain and kinesiophobia is stated in several studies [21,22]. However, we did not identify a significant correlation between pain and kinesiophobia in patients in our study. This situation may have arisen from the fact that the patients did not have severe pain during their evaluation or could be due to different sample sizes. Leeuw et al. found that in patients with chronic musculoskeletal pain, level of pain is related with fear of reinjury and fear of movement [23]. In a study that had been conducted with 2351 RA patients, Lööf et al. concluded that there is a significant correlation between pain and fear-avoidance beliefs of activities. According to the authors, these results may indicate that the fear-avoidance of activity beliefs can play an active role in the transition from acute pain to chronic pain for RA patients characterized by chronic pain. Stress, negative mood, and negative psychological status have been reported to be effective in these results [10].

Depression is a very common symptom of rheumatic diseases. In the literature, studies examining the rate of depression in SLE patients are available. In their study with SLE patients, Ganz et al. reported that 51% of the patients were diagnosed with depression, while Liang et al. reported that they revealed a depression rate of 41% in the total of the patients [24]. In our study, we determined that 53.63% of our patients have mild to very serious depression. These results are consistent with the rates in the literature. The level of depression in our patients, in addition to kinesiophobia, had a significant correlation with fatigue, disease activity, pain, and various subscales of quality of life. Interestingly, although it is considered that depression should affect activity, no significant correlation was found amongst physical activity level and physical activity subscale of quality of life in our patients. Similar to our findings, Munsterman et al. could not find a relationship between physical activity level, low aerobic capacity and depression in patients with RA [25]. While no significant correlation between depression and physical activity was found, a correlation between kinesiophobia and depression was evident. This situation made us think that SLE patients’ fear and avoidance of movement may be associated with psychosocial factors. This is highlighted in a study that was conducted by Wouters et al. [9]. The authors evaluated the physical activity level and perception of physical activity of patients with primary SS in this study. For this, they divided TSK into two subparameters. One of them was pain or injury concerned activity avoidance (“I’m afraid that I might injure myself if I exercise”, etc.), and the other was somatic focus of psychological factors (“My body is telling me that I have something dangerously wrong”). Patients who had a higher somatic focus score were found to have lower physical activity levels and higher mental fatigue. This result confirms that the TSK is a questionnaire that interrogates psychological factors [9]. This may explain the strong relationship between depression and kinesiophobia in our study.

In the general population, physical activity is known to reduce cardiovascular diseases and the risk factors related to them. In addition to that, it also prevents muscle atrophy and osteoporosis in the musculo-skeletal system, it improves posture and has a positive effect on negative mood and stress [26]. The level of physical activity in rheumatic diseases has been demonstrated by several studies to be low. According to these studies, fatigue, pain, morning stiffness, many disease-related symptoms such as psychological disorders may lead to physical inactivity. Demographic and biological characteristics other than disease-related symptoms, mental and emotional status, environmental factors and sociocultural factors are also shown among the factors that hinder physical activity [27]. There are fewer studies investigating the level of physical activity in patients with SLE compared to other rheumatic diseases. In a study conducted by Costanbader et al., physically inactive patients are expressed in 59% of the proportion [28]. Katz et al. concluded that 28% of their patients were inactive in terms of physical activity [29]. Differences of these rates in the literature may be due to the diversity between the study protocols. It may also be due to the evaluation methods used. When we classified patients according to their physical activity levels in our study, we found that 52.17% of them were physically inactive. This result shows similarity with that of the literature. According to our study, physical activity level of patients had an inverse association with kinesiophobia score. However, this relationship was not a statistically significant correlation. This result can be interpreted as that the physical activity status of SLE is not affected much by kinesiophobia. However, different conclusions can be reached with a vast number of patients or with different evaluation methods such as accelerometer which is an objective method. In the literature, the only study assessing avoidance of movement and physical activity level of patients with SLE as stated before is the study by Mancuso et al. [11]. However, this study did not examine the correlation of physical activity level and avoidance. The authors commented that negative beliefs and thoughts may be the cause of physical inactivity. Despite the limited number of SLE-related studies in the literature, there are several studies investigating the relationship between physical activity level and kinesiophobia in rheumatic diseases. In a study of primary SS patients, it was concluded that high kinesiophobia level was associated with low physical activity level [9]. 

There are a number of studies assessing fatigue in rheumatic diseases. In some studies, in patients with AS, fatigue is shown to be the third most common finding after pain and stiffness, and is estimated to affect 53% of patients [30]. However, in patients with RA it has been reported to be between 40% and 80% [31]. In studies performed in patients with SLE, the primary complaint has been reported to be fatigue by 53% to 80% of patients [32]. Fatigue in SLE is shown in various studies to be associated with depression and the ratio of that is higher compared to diseases like multiple sclerosis [33]. In a study with patients with SLE evaluating the relationship between fatigue and pain, a significant correlation was found between the two parameters [34]. Studies that examined the relationship between disease activity and fatigue are controversial. In some studies, a significant correlation was found [35], while no correlation was found in many others [34]. We have obtained the result that fatigue in our patients is associated with depression, pain, and sleep subscale of quality of life. Our results are in line with the results in the literature.

Our study is important because it is the first study to evaluate and classify kinesiophobia in SLE patients. Further investigation on this topic and other studies must be conducted including a control group. As with other rheumatic diseases, for SLE patients’ rehabilitation, exercise is of great importance because of its positive contribution to the musculoskeletal system. However, because rheumatic diseases are chronic, inflammatory diseases which have activation-remission periods, ensuring the patient’s participation in treatment and continuity is of great importance. Evaluation of kinesiophobia in these patients and attempting to overcome if it is present, may contribute to the rehabilitation of these patients.

Using patient-rated scales to evaluate kinesiophobia and other parameters may be a limitation of the study. Number of patients and including only female patients may also be a limitation. Different results may be reached by a larger sample including male and female patients.
